# Metaphor as a cognitive and relational tool for self-narrating experience of addiction: a qualitative-quantitative analysis

**DOI:** 10.3389/fpsyg.2025.1658238

**Published:** 2025-10-03

**Authors:** Daniela Altavilla, Greta Mazzaggio, Valentina Deriu, Stefana Garello, Alessia Vecchi, Ines Adornetti, Alessandra Chiera, Stefano Canali, Francesco Ferretti

**Affiliations:** ^1^Cosmic Lab, Department of Philosophy, Communication and Performing Arts, “Roma Tre” University, Rome, Italy; ^2^Department of Literature and Philosophy, University of Florence, Florence, Italy; ^3^Scuola Internazionale di Studi Superiori Avanzati (SISSA), Trieste, Italy

**Keywords:** addiction, metaphor, self-narrative, identity, mental disorders

## Abstract

**Introduction:**

Metaphors have been acknowledged as crucial for understanding and articulating complex experiences, helping individuals make sense of emotional and social challenges, especially during tough times. In the context of addiction, previous studies have highlighted the potential of metaphorical language to facilitate the expression and comprehension of emotions related to addictive behaviors. However, little research has explored when and what types of metaphors people use in their personal stories about addiction. Aim of current study was to address this gap by analyzing metaphorical language in self-narratives of individuals with addiction.

**Methods:**

Sixty-three participants (37 men and 26 women; age range: 18–65 years) undergoing treatment at public addiction services were recruited. Self-narratives were elicited through a semi-structured interview covering eight addiction-related topics: aspecific desire, definition of addiction, onset of addiction, specific desire/craving, loss of control, relationships with the environment, relapses, and future self-projection. The occurrence of seven metaphorical clusters was identified and quantified: structural, personification, split-self, bodily, movement, ontological, and idiomatic metaphors.

**Results:**

The main findings showed a significantly higher frequency of metaphor use in the definition of addiction compared to all other thematic areas. Moreover, ontological and movement metaphors were especially prevalent in narratives addressing the definition and onset of addiction.

**Discussion:**

These results highlight how individuals with addictive disorders tend to concretize their experience through specific methaphorical patterns—particularly, ontological and movement metaphors. Overall, the use of these metaphor forms appears to provide emotional containment and representational clarity, enabling individuals to express their inner conflicts and emotional ambivalence associated with addiction.

## Introduction

Human beings make sense of their lives through stories. Across disciplines—from narrative psychology to philosophy—there is growing agreement that identity is not a fixed essence but a narrative construction, emerging through the interpretative process of telling, retelling, and reinterpreting one's experiences over time. Several authors argue that identity is built through a narrative process, for example Dennett (1991, 2014) defined the self as the narrative center of gravity, (Bruner 1991, 1996) underlined how, through narration, people can remodel their self and their life story. Similarly, other authors (Gallagher, 2000; McAdams, 1985, 1996, 2001; McAdams and Olson, 2010; McLean, 2017; Ricoeur, 1991) have underlined how the narrative process is fundamental to giving meaning to one's experience, structuring and shaping one's identity over time. In this view, the self is a story in motion, shaped not only by what one lives through, but by how those experiences are represented, structured, and communicated in language.

Within this narrative construction, metaphor can play an important role. Far from being a merely ornamental feature of language, metaphor has been argued to functions as a cognitive and relational tool—a means of grasping, framing, and communicating aspects of experience that are difficult to access through literal language (Lakoff and Johnson, 1980). Especially in moments of disruption, ambiguity, or suffering, metaphor may provide symbolic scaffolding that enables individuals to organize emotional, bodily, and social experiences into coherent patterns of meaning.

Lakoff and Johnson (1980) laid the groundwork for conceptual metaphor theory by illustrating how metaphors function through structured correspondences between two conceptual realms: a concrete or familiar source domain and a more abstract or less tangible target domain. For instance, the metaphor “*illness is a journey*” frames the complex experience of illness through the simpler and more concrete schema of a journey: the patient is a traveler, diagnosis a departure, treatment a path, and recovery a destination (Rossi, 2025; Sopory, 2017). Such metaphorical mappings influence not only vocabulary but also the underlying conceptual frameworks through which illness is understood and navigated in clinical and everyday contexts.

Metaphors also vary in their expressive depth. Some become so familiar that their figurative roots fade from awareness—these are known as *conventional metaphors* (Lakoff and Johnson, 1980; Kovecses, 2010). Others remain *creative or novel*, capable of unsettling expectations, offering new perspectives, and revealing personal truths (Gibbs, 2011; Cameron, 2003). These novel metaphors can be especially potent in personal narratives, where speakers attempt to convey affective experiences, internal conflict, or identity shifts. In this way, metaphor can serve not only as a linguistic form but also, in some contexts, as a mechanism for symbolic transformation, a mode of reconfiguring experience (Semino, 2008), that can also influence how we conceptualize important societal issues (Thibodeau and Boroditsky, 2011).

### Metaphors in mental disorders

Metaphor occupies a crucial, if often underexamined, place in clinical discourse. While traditionally viewed through the lens of cognitive deficit—particularly in psychiatric contexts where patients are analyzed for their difficulties in metaphor understanding—metaphor should also be understood as a *tool* for communication, relational connection, and narrative identity work (Garraffa and Mazzaggio, 2025). Clinical research has historically centered on difficulties in metaphor comprehension, especially in schizophrenia, where impairments in figurative language processing have been well-documented (Rapp, 2009; Titone et al., 2002; Iakimova et al., 2005). Individuals with schizophrenia frequently display a cognitive style referred to as *concrete thinking* (Goldstein, 1944), marked by a preference for literal interpretations and difficulty grasping idioms (Perlini et al., 2018), proverbs (Thoma et al., 2009), and metaphors (de Bonis et al., 1997; Mashal et al., 2013; Perlini et al., 2018; Schettino et al., 2010). Neuroimaging studies suggest that these deficits do not reflect a deterioration of semantic knowledge—that is, the mental store of word meanings and conceptual associations—but rather impairments in inhibiting literal interpretations and integrating contextual cues essential for figurative understanding (Mashal et al., 2013; Rapp et al., 2007; Titone et al., 2007).

However, the overwhelming focus on comprehension has obscured a diverse view of metaphor as something *used* by patients, rather than merely misunderstood. Spontaneous metaphor production—arguably a more ecologically valid expression of figurative language—is far less studied. Yet recent evidence indicates that individuals with schizophrenia and related conditions may generate rich, even inventive metaphors, though often atypical or idiosyncratic (Billow et al., 1997; Kuperberg, 2010; Despot et al., 2021). This suggests a more complex relationship between figurative language and psychopathology than deficit models allow.

### Shifting focus from decoding to metaphorical production

Reframing metaphor, figurative language should not be reduced to a problem of decoding; it can indeed be situated within a social-cognitive framework where metaphor functions as a therapeutic tool for meaning-making, emotional regulation, and relational engagement (Radley and Chamberlain, 2001; Kirmayer, 1992). Beyond its cognitive and communicative dimensions, metaphor also functions as a form of psychological coping—a symbolic strategy through which individuals manage emotional pain, social stigma, and disruptions in identity associated with mental illness and psychological distress (White, 2002; Malvini Redden et al., 2013). By giving shape to otherwise diffuse or overwhelming affective states, metaphor allows patients to externalize their experiences and render them into forms that are symbolically structured and emotionally containable (Demjén, 2014). In this capacity, metaphor acts as a protective buffer against existential threat, enabling individuals to gain distance from their suffering, to reframe its meaning, and to imagine alternative possibilities (Semino, 2008; Cameron, 2003). At the same time, metaphor provides a medium through which people can construct the significance of their illness, helping them to translate unfamiliar and fragmented experiences into forms that are communicable and meaningful within a shared framework. Specifically, they perform three main functions: *naming*, by giving patients a way to articulate internal processes and personal meanings of illness; *framing*, by shaping experiences through specific affective perspectives that aid self-understanding; and *changing*, by offering new ways of relating to the illness without complete identification (Ervas, 2024, 2025).

For example, in the context of depression, metaphorical language often clusters around themes of *darkness, heaviness, descent*, and *entrapment* (Charteris-Black, 2012; McMullen and Conway, 2002). Other common metaphorical frameworks include spatial orientation (*up/down*), *containment, journey, enemy*, and even *malfunctioning machinery*, reflecting the embodied and oppressive nature of depressive states (Semino, 2008; Fahlenbrach, 2017). These patterns emerge not only in clinical interviews but also in naturalistic settings. (Coll-Florit et al. 2021a,b), analyzing mental health discourse in online blogs, found that both patients and professionals used metaphors to frame their understanding of disorders such as depression, schizophrenia, bipolar, and obsessive-compulsive disorders. Patients, however, tended to produce more metaphors grounded in first-person experience about their mental disorder, while professionals used more operational or treatment-oriented metaphors. Despite diagnostic differences, the most prevalent metaphors among patients were consistent: illness as a *journey*, a *war*, a *living entity*, a dark *place*, or a *container*, often accompanied by a pervasive *sense of fragmentation* or a *split self* . Similarly, in a study of dementia blogs, (Castaño 2023) found that individuals diagnosed with early-onset dementia consistently used *journey* and *relational* metaphors to frame their psychological needs—particularly autonomy, competence, and relatedness. These metaphors were not merely expressive but functioned to maintain coherence and assert personhood amid cognitive decline.

For individuals with psychosis, characterized by altered experiences of self, such as fragmentation (Davidson et al., 2004), the rebuilding one's sense of self is an important part of the recovery process (Andresen et al., 2003). In this regard, several studies have investigated metaphors in this context. In their systematic review, Mould et al. (2010) observed that metaphors often serve two complementary functions: *ontological metaphors*, which provide bounded and entity-like imagery, tend to support the consolidation of self, helping individuals to stabilize and ground their sense of identity; while *orientational metaphors*, grounded in spatial movement (up/down, in/out, forward/backward), assist in transitioning the self, enabling patients to narrate progress and envisage change. Since both consolidation and transition are central processes in recovery, Mould et al. (2010) argue that these types of metaphor represent basic linguistic and cognitive resources that not only aid in the explanation of subjective experiences, but can also be therapeutically mobilized to reconstruct identity and promote growth.

Given this evolving understanding, it is essential for clinical research to move beyond static models of comprehension impairment and focus on metaphor production as an active process of self-articulation, attending to how metaphor *production* functions in the construction and negotiation of selfhood—particularly in populations where identity is vulnerable, unstable, or contested. This can be especially relevant in the context of addiction, where metaphor might emerge not only as a stylistic device but as a vital conduit for expressing ambivalence, inner division, and the ongoing struggle for coherence amid fragmentation.

### Metaphorical production in addiction

Addictive disorders include various conditions marked by persistent substance use or repeated behaviors that negatively affect the person's social and personal life, leading to significant suffering (American Psychiatric Association, 2013). This condition is deeply marked by narrative rupture, identity conflict, and the pervasive social stigma. These forces shape not only individuals' psychological and physical wellbeing but also their ability to articulate coherent life stories (Canali et al., 2021). Metaphors such as “*being in a cage*,” “*fighting a demon*,” or “*falling into a hole*” structure how individuals and societies conceptualize compulsion, agency, relapse, and recovery (Montagne, 1988; Marlatt and Fromme, 1987; Malvini Redden et al., 2013). Another recurring theme in addiction narratives is the experience of a fragmented or divided self—simultaneously desiring and resisting—a state that disrupts perceived agency and coherence (Dill and Holton, 2014; Levy, 2006; Lewis, 2015). Indeed, addiction is often described as a force external to the self, experienced as something alien or invasive, contributing to dissociative dynamics in which actions feel detached from volition. Scholars have linked this loss of self-integration to diminished self-efficacy, where cravings or compulsive behaviors are seen as emanating from an “other” within (Altavilla et al., 2020; Canali et al., 2021; Keane, 2002; Reith and Dobbie, 2012; Schluter and Hodgins, 2019). In response, narrative becomes a critical site for self-repair and symbolic re-integration. Through storytelling, individuals attempt to reassert continuity, reclaim agency, and make emotionally and socially intelligible sense of their experience (Adornetti and Ferretti, 2021; Benítez-Burraco et al., 2023) and metaphor can occupy a specific role in this process. It might indeed structure how people frame some key aspects of addiction—like desire, compulsion, relapse, recovery—by offering conceptual architecture to interpret complex and often contradictory internal states. Indeed, individuals have been indicated to use metaphors to express their oscillation between control and loss, hope and despair, passivity and agency, as well as to mediate the tensions between personal suffering, stigma and social judgment (Biong et al., 2008; Malvini Redden et al., 2013). Marlatt and Fromme (1987) described as the “abstinence violation effect,” when a single lapse can lead to a collapse of perceived agency and identity continuity, often metaphorically experienced as *falling, drowning*, or *spiraling*. Thus, narratives allow speakers not only to recount what happened, but to explore how it felt, and what it means, in light of shifting self-conceptions and societal expectations (Pennebaker, 2000). This might be particularly true in the case of metaphors, which, as shown by Lakoff and Johnson (1980), provide powerful cognitive and relational tools for structuring and conveying lived experience.

Although metaphor is pervasive in how addiction is described, empirical research explicitly analyzing metaphor use within addiction narratives remains limited—though this landscape is beginning to shift. Early qualitative work laid important foundations. For instance, Malvini Redden et al. (2013) examined the metaphorical language of individuals undergoing methadone treatment across different racial and ethnic groups. Participants described their drug use through empowered, agentic language (e.g., “*taking*,” “*choosing*”), while recovery was often framed using passive or externalized imagery (e.g., “*rescued by an angel*,” “*trapped by liquid handcuffs*”). Treatment itself emerged as an ambivalent metaphor: simultaneously salvation and imprisonment. These metaphors captured not just affective nuance, but also political tensions surrounding control, dependence, and autonomy.

As Malvini Redden et al. (2013) claim, metaphors not only illustrate the communicative depiction of experience but essentially act as a shorthand for cognition. Addictionally, Povozhaev (2014)—through a rhetorical case study of metaphor use in real-time clinical interactions at a methadone clinic—underlies that patients often framed addiction as an embodied, emotionally fraught illness experience whereas the clinician favored a biomedical disease model. This structural misalignment in metaphor use shaped conversational flow and therapeutic rapport, suggesting that metaphor is not only a narrative strategy but a co-constructed diagnostic and relational tool.

While Malvini Redden and Povozhaev focus on institutional and clinical contexts, other studies have explored metaphor in more introspective, autobiographical narratives. Shinebourne and Smith (2010), using interpretative phenomenological analysis, investigated the metaphorical landscapes of individuals with alcohol dependency. These included addiction as affliction and support, recovery as growth, and addiction and recovery as a journey. Moreover, some participants in the study used metaphors to avoid dealing with painful emotions, describing their feelings as things to be *blocked, blacked out, killed*, or *boxed*. Other authors (Montagne, 1988; White, 2002) note that such metaphors are common in discussions about addiction, suggesting that responsibility is often displaced onto non-human forces—*demons, beasts*, or *sirens*—revealing a mythical or archetypal framing. These metaphorical configurations allow for the externalization of guilt, and the mythification of personal struggle, which can both reduce shame and clarify experience. In accordance, Gyuro (2016) identified several metaphor classes present in drug users' language—including those of *flight, depression, transcendence*, and *journey*—suggesting that they are structured to create a system in which the affective, cognitive, and behavioral dimensions—which are often incoherent and fragmented in addiction—become mutually interconnected. Moreover, Marlatt and Fromme (1987) also emphasizes how metaphors can structure the very possibility of relapse prevention, offering individuals symbolic resources to anticipate risk and navigate emotional terrain.

Taken together, these studies seem to demonstrate how metaphor can function as a symbolic resource for articulating divided agency and reconstructing fractured self-narratives. They often emerge in accounts of recovery, where they help articulate the shift from a fragmented, stigmatized self toward a re-authored identity grounded in survivorship and self-understanding (Shinebourne and Smith, 2010).

While these pioneering studies illuminate important aspects of metaphor use in addiction, there remains a pressing need for more systematic investigations. Specifically, research is lacking on how different types of metaphors are distributed across various thematic domains within individuals' self-narratives, and how these metaphorical patterns contribute to the ongoing construction of identity and meaning in addiction and recovery.

### The present study

The present study seeks to address this gap by analyzing metaphorical expressions in the self-narratives of individuals with addictive disorders, elicited through a semi-structured interview. The interview explored eight thematic areas which—as highlighted by several authors (e.g., Alksne et al., 1967; Feltenstein et al., 2021; Koob, 2011; Koob and Volkow, 2010)—characterize the behavior and the experience of addiction: aspecific desire, definition of addiction, onset, desire, loss of control, relationship with the environment, relapse, and future orientation. Within these narratives, we identified and categorized metaphors into seven clusters: structural, personification, split-self, bodily, motion, ontological, and idiomatic.

Rather than testing predefined hypotheses, this study adopts an exploratory approach. To shed light on how metaphor serves as a dynamic tool for meaning-making, emotion and craving regulation, identity negotiation, and the articulation of agency in addiction narratives, we seek to identify patterns of metaphor use both within and across thematic areas. This method stresses the symbolic work through which people create cohesive and agentive self-stories despite the difficulties presented by addiction, acknowledging the complexity and individuality of metaphorical expression.

In sum, the present study aims to advance knowledge of the relationship between language, identity, and recovery by charting the distribution and purpose of various metaphor clusters in addiction self-narratives. Since exploring patients' subjective experience through their first-person narratives—thereby analyzing what they say explicitly and what they communicate implicitly—is a way to understand the patient's inner world beyond theory, the findings of the present study could guide clinical practice, encouraging empathetic and sensitive approaches to the subjective dimension of addiction.

## Methods

### Participants

A total of 63 participants (37 men and 26 women; age range: 18–65 years) diagnosed with addictive disorder according to criteria of the *Diagnostic Statistical Manual of Mental Disorders* (DSM-5; American Psychiatric Association, 2013): 2 smokers, 5 cannabis, 18 heroin, 5 cocaine, 2 alchol, 19 poly-substance, 11 gambling. Patients were under treatment and recruited from Italian public Service for Addiction at Trieste, Udine, Gorizia, Pordenone, Tolmezzo e Gemona del Friuli.

Participants signed the consent form for the participation to the study and for the treatment of the data. The Ethical Committee of the Scuola Internazionale di Studi Superiori Avanzati—SISSA-Trieste approved the study. Committee approval is in accordance with ethical guidelines detailed in the 1964 Helsinki Declaration or any of its succeeding amendments.

### Procedure

This study adopts a quali-quantitative approach grounded in semi-structured interviews, designed to explore the subjective and metaphorical representation of addiction among individuals in treatment. The interview protocol was structured to investigate eight major thematic areas that span both experiential and conceptual dimensions of addiction: (1) aspecific desire, (2) definition of addiction, (3) onset of addiction, (4) specific desire/craving, (5) loss of control, (6) relationships with the environment, (7) relapses, and (8) future self-projection.

These macro-areas were detected through clinical observations and theoretical models of addictive behavior (e.g., Alksne et al., 1967; Feltenstein et al., 2021; Koob, 2011; Koob and Volkow, 2010). Each was operationalized through targeted, open-ended questions aimed at eliciting detailed first-person narratives that could reveal metaphorical language, emotional tones, and behavioral patterns.

In particular, the topic areas were defined as follows:

1) *Aspecific Desire:* participants were asked to describe experiences of intense non-substance-related desires (e.g., food, sex) with potentially negative consequences. Participants were asked to imagine such situations and describe their cognitive and emotional responses: “Can you think of a time when you felt a strong desire for something, but knew it could be harmful?” and “What usually goes through your mind or body in such a moment?”. Participants were also asked to reflect on how these desires typically arise and how they attempt to regulate or respond to them.
2) *Definition of Addiction:* respondents were asked to explain addiction from their personal point of view: “How would you define addiction to someone who knows nothing about it?” Additional prompts explored their agreement with the medical model of addiction (e.g., “Do you see addiction as a disease?”).
3) *Onset of Addiction:* this section explored autobiographical narratives related to the origins of addictive behavior, they were asked: “What do you think led to the beginning of your addiction?” and “Were there specific situations or emotional states that played a role?”.
4) *Specific Desire/Craving:* participants were encouraged to describe the experience of craving: “What happens in your mind and body when you feel the urge to use [the substance]/gamble?”. Further questioning explored the contextual cues that might amplify desire: “Were there places, situations, or conversations that made the craving stronger?”.
5) *Loss of Control:* Participants were asked to describe the moments preceding compulsive use: “What seemed to happen just before you lost control?” and “Was there an internal conflict between your desire and your will to resist?”. These questions aimed to surface the transition from intention to impulsive action, as well as the participant's level of awareness during this shift.
6) *Relationships with the Environment:* the interview examined the perceived impact of familial, social, and environmental factors on the development and perpetuation of addiction. Participants responded to prompts such as: “What impact do you think your family, friends, or social environment had on your addiction?” and “How did they react when they found out about it?”.
7) *Relapses:* Participants discussed their experiences with relapse, including precipitating events, emotional responses, and reflections on the cyclical nature of recovery and regression (e.g., “Since you began treatment, have you experienced any relapses? What triggered them?”).
8) *Future Self-Projection:* Respondents reflected on their expectations and hopes for the future, especially in terms of abstinence maintenance, life goals, and self-redefinition, with questions like: “How do you see your life in the future—not just regarding addiction, but more broadly?”.

All interviews were conducted by trained clinicians in a therapeutic setting. Sessions were recorded in audio format and subsequently transcribed verbatim to preserve the integrity of the narratives.

In the initial phase of analysis, the full corpus of each interview was manually segmented into the eight aforementioned thematic areas. In the second phase, each thematic segment was divided into discrete utterances for fine-grained linguistic and metaphorical analysis. Utterances segmentation was carried out by adapting the method by Marini et al. (2011a,b), in order to calculate the percentage of metaphorical occurrences depending on the number of utterances. They were defined following the semantic and grammatical criteria (Adornetti et al., 2022, 2024; Ferretti et al., 2018; Marini et al., 2011a,b). Specifically, according to the semantic criterion, an utterance corresponds to an homogeneous unit of information, i.e., a proposition, which corresponds to a semantic unit comprising a main predicate, its argument, and any embedded predicates and associated arguments (e.g., the excerpt “I started using when I was 15 because I felt alone and it help me deal with daily life. Then I lost control” can be segmented into two distinct utterances “I started using when I was 15 because I felt alone and it help me deal with daily life. /Then I lost control”); according to the grammatical criterion, a set of words can be considered an utterance when, in absent to propositional violations (semantic criterion), it constitutes a grammatically complete sentence, including subordinates (e.g., “I decided to enter treatment because I couldn't live like that anymore” can be considered as a single utterance, while “I would steal from my wife to buy drugs but she found out and I had to leave home” can be segmented in three different utterances “I would steal from my wife to buy drugs / but she found out /and I had to leave home”).

### Metaphorical cluster

In this study, we adopt a definition of metaphor inspired by cognitive linguistics (Lakoff and Johnson, 1980) but operationalized through an interpretative and corpus-driven procedure adapted from Shinebourne and Smith (2010). Unlike other methodologies as the Metaphor Identification Procedure (MIP; Steen and Pragglejaz Group, 2007), which adopt the lexical unit as the unit of analysis and require word-by-word coding, our analysis was based on the level of the utterance. Within each utterance, we identified metaphorical expressions that were contextually salient to the participant's meaning-making. This shift in the unit of analysis reflects our focus on narrative and clinical significance rather than exhaustive lexical annotation, while still being compatible with the general principle of contrasting contextual and basic meanings. We focused in particular on creative or “live” metaphors, namely those expressions that actively structured, framed, or transformed participants' accounts of their experiences. The guiding principle was always contextual relevance: we asked whether, in the given narrative, the metaphor provided the speaker with a non-literal but meaningful way of articulating concepts related to their lived experience of dependence and beyond. Accordingly, we excluded “dead” or fully lexicalized metaphors whose figurative origin is no longer perceived by speakers and which did not contribute to the symbolic or experiential work of the narrative. Conversely, idiomatic or conventional expressions were retained when they clearly carried expressive or symbolic force in context.

Metaphors were thus identified through an iterative, corpus-driven procedure. Each interview transcript was first read independently by pairs of researchers, who individually highlighted candidate metaphorical expressions. As in previous studies (e.g., Cameron and Maslen, 2010; Mathieson et al., 2015; Qiu et al., 2024), metaphors were identified in single words as well as in larger language units such as phrases and sentences. Coders then met to compare their annotations, and only expressions agreed upon by all were retained. Inter-rater reliability was high (*r* = 0.92, *p* < 0.001), and discrepancies were resolved through consensus discussion.

As the coding progressed, metaphors identified across interviews were compiled into provisional tables. Through repeated group discussions, these tables were gradually organized into clusters reflecting conceptual similarities. Once a preliminary set of clusters had been agreed upon, the team revisited the interviews and re-examined the metaphors in this light, refining both the boundaries of the clusters and the allocation of examples. This iterative process allowed us to balance inductive grounding in the data with theoretical sensitivity to the broader literature on metaphor and addiction. In the final stage, seven clusters were retained: *Structural metaphors* involve mapping one complex concept onto another (e.g., “*addiction is a trap*”) and are often used to explain abstract phenomena. *Personification metaphors* attribute human qualities or agency to non-human elements (e.g., “*the drug calls me*”), enabling individuals to externalize their internal experiences. The *split-self cluster* includes metaphors that reflect a divided internal state or conflict between parts of the self; these are particularly relevant in narratives of craving and control (e.g., “*a part of me wanted it*”). *Bodily metaphors* draw directly on sensorimotor or somatic language (e.g., “*a hole in my chest*”), grounding emotional states in physical experience. *Motion metaphors* represent addiction and recovery as directional movement (e.g., “*falling back*,” “*moving forward*”), which are often used to narrate change over time. *Ontological metaphors* frame addiction as a bounded object or entity (e.g., “*my addiction is a crutch*”), providing a way to contain and conceptualize challenging experiences. Finally, *idiomatic metaphors* include fixed or culturally embedded phrases (e.g., “*hit rock bottom*”) which have acquired figurative meanings through their conventional usage.

The percentage of each metaphorical cluster was then carried out across the eight thematic areas after this process. This allowed for a cross-sectional analysis of metaphor use in different narrative contexts, combining quantitative mapping of metaphorical patterns with qualitative interpretation of their function and meaning. This methodology enabled a multi-layered understanding of how individuals use metaphor to construct and communicate their experiences of addiction and recovery.

### Statistical analysis

A repeated-measures ANOVA was conducted with Thematic Area (eight levels: aspecific desire, definition of addiction, onset, desire, loss of control, relationships with the environment, relapses, self-projection into the future) and Metaphorical Cluster (seven levels: structural, personification, split self, bodily, movement, ontological, idiomatic) as within-subject factors.

For *post-hoc* analysis Bonferroni test was applied. All analysis were performed with Statistica (StatSoft Inc., v.7, 2004).

## Results

**Figure 1:**
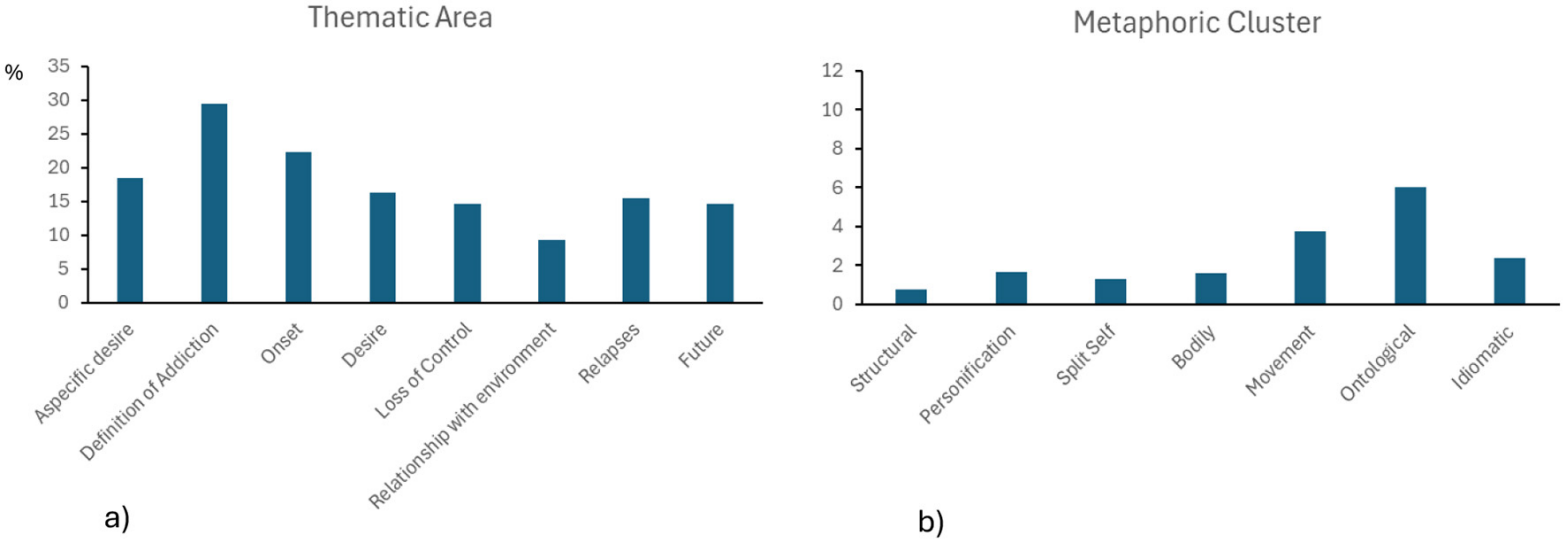
Average percentage of metaphors in each thematic area **(a)** and average percentage of metaphors for each metaphor cluster **(b)**.

A repeated-measures ANOVA revealed a significant main effect of Thematic Area on metaphor frequency [*F*_(7, 434)_ = 6.84; *p* < 0.001], where the percentage of metaphors was significantly higher in the *definition of addiction* than *aspecific desire* (*p* = 0.022), *desire* (*p* = 0.002), *loss of control* (*p* < 0.001), *relationships with the environment* (*p* < 0.001), *relapses* (*p* < 0.001) and *self-projection into the future* (*p* < 0.001); additionally, the percentage of metaphors in the *onset* area category was significantly higher than in *relationships with the environment* (*p* = 0.002; Figure 1a). A significant main effect of Metaphorical Cluster [*F*_(6, 372)_ = 31.25; *p* < 0.001] was also found. *Post-hoc* analysis showed that the percentage of *movement metaphors* was significantly higher compared to *structural, personification, split self* and *bodily metaphors* (all *p* < 0.001); the percentage of *ontological metaphors* was significantly higher compared to *structural, personification, split self* , *bodily, movement* and *idiomatic metaphors* (all *p* < 0.001); and the percentage of *idiomatic metaphors* was significantly higher compared to *structural metaphors* (*p* < 0.001; Figure 1b).

**Figure 2:**
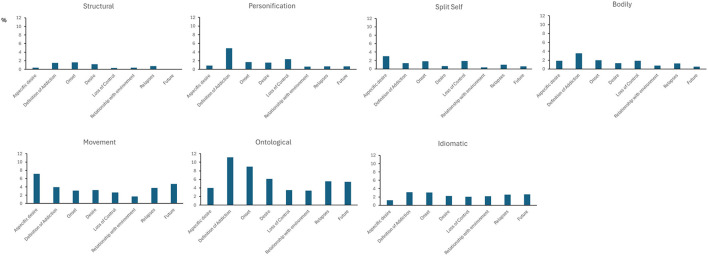
Average percentage of metaphors (y-axis) in each metaphor cluster for each thematic area (x-axis)

Beyond the main effects, a significant interaction between Thematic Area and Metaphorical Cluster was observed [*F*_(42, 2604)_ = 2.69; *p* < 0.001], indicating that certain types of metaphors were more prevalent in specific thematic contexts (Figure 2). Paired comparisons showed a significantly higher percentage of *ontological metaphors* during the narration of the *definition of addiction* compared to the narration of the *aspecific desire, loss of control, relationships with the environment, relapses* and *self-projection into the future* (all *p* < 0.001). A similar pattern was found in the *onset area*, where *ontological metaphors* outnumbered those found in the narration of the *aspecific desire* (*p* = 0.007), *loss of control* (*p* < 0.001) and *relationships with the environment* (*p* < 0.001). Additionally, *movement metaphor*s were significantly more frequent in the *onset* section than in *relationships with the environment* (*p* < 0.001).

**Figure 3:**
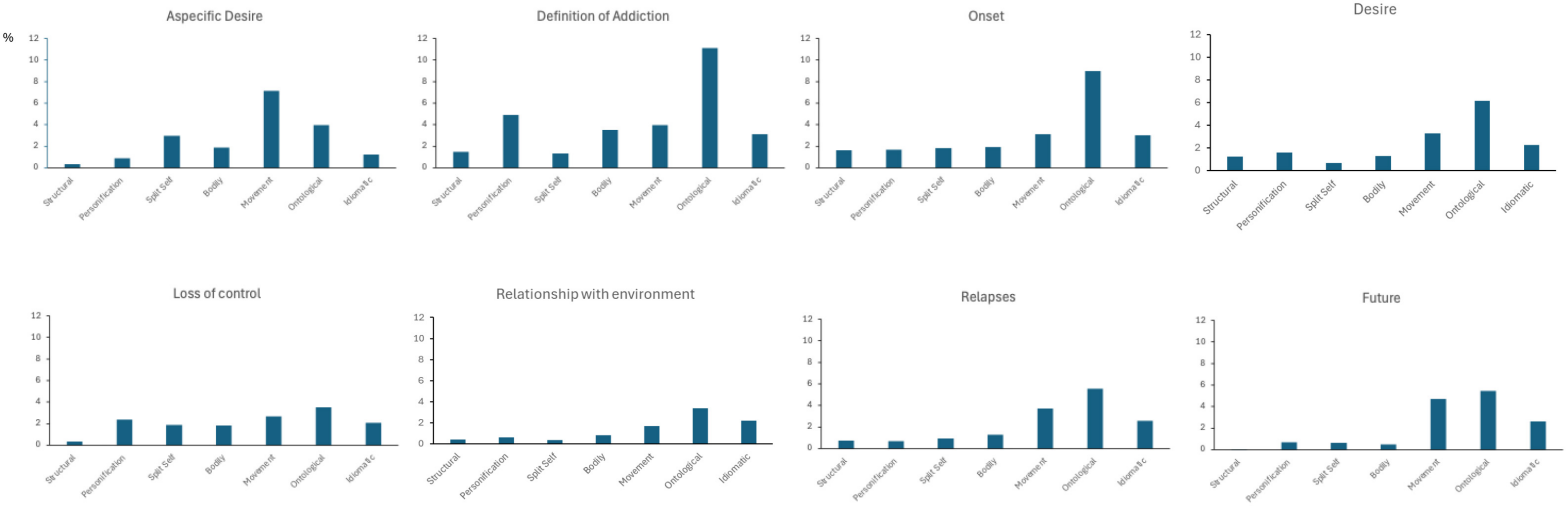
Average percentage of metaphors (y-axis) in each thematic area for each metaphor cluster (x-axis).

Further comparisons across metaphorical clusters within each thematic area revealed distinct patterns (Figure 3). In the narration of *aspecific desire, movement metaphors* occurred significantly more often than *structural, personification, bodily*, and *idiomatic metaphors* (all *p* < 0.001). Both the *definition of addiction* and *onset* narratives showed a consistent dominance of *ontological metaphors* over all other types, including *structural, personification, split self* , *bodily, movement*, and *idiomatic metaphors* (all *p* < 0.001). In the *desire* area, *ontological metaphors* were again significantly more frequent than *structural* (*p* = 0.010), *personification* (*p* = 0.041), *split self* (*p* < 0.001), and *bodily metaphors* (*p* = 0.014).

During the narration of *relapse, ontological metaphors* surpassed *structural* (*p* = 0.014), *personification* (*p* = 0.012), and *split self metaphors* (*p* = 0.037). Finally, in the *self-projection into the future* area *ontological metaphors* remained dominant, significantly outnumbering *structural* (*p* < 0.001), *personification* (*p* = 0.017), *split self* (*p* = 0.015), and *bodily metaphors* (*p* = 0.009). Additionally, in this same section, *movement* metaphors occurred more frequently than *structural metaphors* (*p* < 0.001).

## Discussion

The current research aims to study the metaphors found in stories about addiction by combining qualitative and quantitative analysis. Personal stories were collected through semi-structured interviews that explored eight key areas related to addiction: aspecific desire, definitions of addiction, onset, desire, loss of control, relationships with others, relapses, and projection into the future. During the interviews, seven metaphorical clusters were recorded: structural, personification, split-self, bodily, movement, ontological, and idiomatic.

The main findings showed a significantly higher percentage of metaphors in the definition of addiction than in all other thematic areas, as well as a higher use of ontological and movement metaphors. Specifically, the interaction effect revealed a higher percentage of ontological metaphors in the definition and onset of addiction, desire, relapse and self-projection into the future; a higher percentage of movement metaphors was found in the onset, as well as in aspecific desire and self-projection into the future.

### Metaphors as cognitive representations of addictive experience

As Mould et al. (2010) and others have argued, ontological and movement metaphors serve as fundamental building blocks of conceptualization, reflecting basic structures of human experience. Their frequent appearance across participants' narratives suggests that these metaphor types offer simple, intuitive frameworks for expressing complex psychological states—particularly when more abstract or symbolic resources may be limited.

Regarding ontological metaphors, in the present study the most common were:

- Definition of addiction*: crutch, refuge, prison, bypass the brain, insurmountable problems, when you're inside, you lock, yourself in addiction, is a mechanism, you cling to substances, stay in the bubble, a vortex, I have burned the most beautiful years*
- Onset: *I have crumbled, closed in on myself, a spring has been triggered, I try it and you're in, a world has opened, shelter, there was always something in my head, my baggage of troubles, it was a dark time in my life, mechanism, desire is a force that becomes tentacular, dark side, I have burned my life, The Land of Fun*
- Desire: *plug the pain, I see myself as inside a gear, game was the refuge, mental relaxation, dream, have that openness you knew only gave you ecstasy, It had eaten everything!, I felt my world, my thoughts fall on me, the only desire was to reset, you're connected to the game, as refuge, parallel world, prison, close myself off from the others*
- Relapse: *every time I knew a piece of me, every time I returned to my cradle, to a safe sea, take back your little stamping, relief valve, I cut out a space that is only mine, shelter, put the pieces of my life together, my head was unfortunately stuck in that world, cut the crap, building up life, hide them under the carpet, empty days, it's a double-edged sword, bubble, prison, still problems inside, stay inside your world, break the daily routine, the rhythms, you feel that lack the pieces, the bricks that hold up the structure*
- Self-projection into the future: *walk without crutch, reborn as La Fenice, I see it rosy, consolidate the love, slowly build up, I don't see her gloomy, I'm getting a little bit of life back in my hand, cut out a space for me, don't fall into oblivion, leave a positive legacy, building a better foundation, do not put too much meat on the fire*.

As Ervas (2025) argues, “in contexts of illness where patients face an epistemic void, metaphors operate as symbolic tools of translation, enabling individuals to convey what is otherwise ineffable by highlighting the aspects of their illness they find most important to share. Specifically, “the “epistemic void” is linked to the absence of prior lived experience to draw upon in order to make sense of one's current condition and, at the same time, to the lack of vocabulary with which to name that condition. A person's experiences can be infinite, even in qualitative terms, whereas our vocabulary is finite: it is therefore not surprising that words are missing precisely for those experiences that are unfamiliar. However, the “epistemic void” also stems from the failure of others to attribute meaning, a shared meaning provided by the community, which is indispensable for the construction of knowledge” (Ervas, 2025, p. 72, *English is ours*).

Ontological metaphors, in particular, have seen to enable abstract concepts, such as activities, emotions, or ideas, to be represented in concrete and physical representations. Indeed, in this study, it appears that people with addiction predominantly represent themselves and communicate their experience in such concrete terms. Given the inherent complexity of subjective experience, translating internal states into tangible, sensory language seems to enhance communicability by reducing ambiguity and facilitating a clearer exchange of meaning with the interlocutor.

In line with the claims of Malvini Redden et al. (2013), the metaphors seem to act as a shorthand for cognition. As a basic metaphor of language, the ontological metaphor allows for a greater immediacy in the sharing of experiences. For example, when a patient compresses the complexity of her/his experience of addiction by representing it as a “*crutch*” or a “*refuge*,” she/he elicits the imagination and semantic knowledge of the interlocutor about the shared meaning that crutch (support) and refuge (environment protected from danger) can have. According to Ricoeur (1978, p. 144) “the vividness of such good metaphors consists in their ability to ‘set before the eyes' the sense that they display,” thus evoking their imaginative dimension (Shinebourne and Smith, 2010).

The prevalent use of ontological metaphors may be a simple and basic way to establish an intersubjective relationship with others when one lacks high-order cognitive tools, such as the ability to mentalize and imagine, that enable greater affective processing (Bromberg, 1998, 2006; Deriu et al., 2024; Fonagy and Target, 1997; Schoenbaum et al., 2016). Indeed, poor emotional regulation is a key trait of this disorder, affecting the intensity and quality of emotions (Jurist, 2005; Savov and Atanassov, 2013). People with addictions struggle to manage strong feelings of anxiety and depression, which affects their ability to make decisions and plans. This leads to social difficulties, emotional distress, and impulsive behavior. Additionally, constant suppression of emotions can result in a condition where they are perceived as physiological assaults, creating vicious cycles such as “I am afraid to be afraid” (Savov and Atanassov, 2013, p. 2). Indeed, several authors (e.g., Khantzian, 1985) agree that addiction often occurs when people try to “self-medicate.” This means that individuals may “*fall*” in addition to cope with pain, stress, or emotional issues. In the light of these considerations, the prevalence of ontological metaphors to define dependence (e.g., “*crutch*” or a “*refuge*”) found in the present research suggest that patients seek comfort and relief from their problems through the object of addiction.

### Metaphors of ambivalence and loss of control

Another aspect of the patients' experience that can be seen as representing their emotional dysregulation and that can also be detected through metaphors is their internal conflict and ambivalence. Specifically, people describe their addiction and narrate their experience of its onset and desire in both positive—*a beautiful world, crutch, refuge, the land of toys, dream, openness*—and negative terms—*prison, dark world, nightmare, tentacled force, closure*. These findings are consistent with previous studies (Malvini Redden et al., 2013; Shinebourne and Smith, 2010), which identified metaphors associated with ideas of affliction and support in how individuals with addiction described their condition and its treatment.

With regard to the movement metaphors, they seem to be another building block of language, akin to the ontological metaphors. In the present study, movement metaphors were prevalent when patients were narrating themes such as aspecific desire, onset, and self-projection into the future. The following are illustrative examples:

- aspecific desire: *I go toward, the peak rises, down, I let go, I try to divert the desire, get out of some lines, go and look for relief, I carry on, I throw myself on that behavior, I let myself slide down, I let myself go, I let myself be carried away, I lose myself*
- onset: *just want to get there, I let myself go, I keep going, it went through ups and downs, a vortex that is difficult to get back up, difficult to get out of, I don't like to rush through life*
- self-projection into the future: *I'm stuck and I'm always afraid to take that step, I have to take small steps, keep going what I'm doing, I keep going, I'm in a loop, I don't want anything to stop me anymore, I never stopped*.

Participants often used movement metaphors to convey their experience of surrendering to addiction. Expressions of “*letting go*” were commonly associated with a perceived loss of control, likely driven by the impulse to self-medicate and escape from overwhelming internal states that are difficult to regulate. This act of psychological release, however, is frequently followed by an intense struggle to break free from the addiction and to re-establish alternative regulatory mechanisms. The resulting emotional turbulence often manifests in persistent mood swings and alternating emotional highs and lows. Although the results are not significant, some patients describe this passivity and loss of control through personification metaphors, when they define dependence and the phase in which they perceive control as an external entity that takes over them. Through such metaphors, individuals appeared to defensively distance themselves from their condition—externalizing the addiction rather than recognizing the vulnerable aspects of the self that seek relief from psychological fragmentation. In doing so, they may further diminish their sense of self-efficacy, agency, and responsibility, reinforcing the cycle of dependence.

### Metaphors in therapeutic and relational contexts

From a therapeutic standpoint, a growing body of research emphasize the importance of metaphor as a communicative tool within clinical settings (e.g., Boone and Bowman, 1996; Cirillo and Crider, 1995; Kopp, 2013; Martin et al., 1992; Shkëmbi and Treska, 2024; Siegelman, 1993; Tay, 2016). Metaphors can be intentionally employed to reframe illness in ways that influence patients' understanding, engagement, and sense of identity. Furthermore, Lyddon et al. (2001) demonstrate that clinicians' sensitivity to patients' metaphorical language fosters therapeutic alliance and empathy. The present study adds to this discourse by showing that individuals with addiction tend to communicate their experiences predominantly through ontological and movement metaphors. These metaphors appear to serve a concrete representational function, possibly reflecting limited capacity for abstraction, imagination, or mentalization. In such cases, metaphor might become not only a stylistic device but a necessary means of emotional articulation. Indeed, as Shinebourne and Smith (2010) and Biong et al. (2008) show, metaphors in narratives of addiction and mental health crises are often not only expressive, but transformative-acting as bridges between chaos and coherence, pain and meaning, disempowerment and agency.

Within the therapeutic relationship, the ability to recognize and elaborate on internal states often emerges through the co-construction of meaning between patient and clinician. When clinicians engage with patients' metaphorical language—or introduce new metaphorical framings—they can help patients re-evaluate entrenched beliefs and develop more adaptive narratives of illness and recovery (Ervas et al., 2016).

In this vein, recent scholarship has extended this inquiry from discourse analysis to encompass therapeutic intervention. For example, Mohamed et al. (2024) conducted a quasi-experimental study to evaluate the effectiveness of metaphor therapy for individuals with substance use disorders. Participants took part in six sessions with metaphorical stories to change their irrational beliefs about drug use. Results showed that the metaphor group had lower irrational belief scores and stronger negative attitudes toward substance use compared to the control group both the post-treatment and the follow-up stage. Future research could create intervention protocols to enhance patients' creative skills based on these findings. These protocols might include guided metaphor development, metaphor substitution, and narrative reconstruction strategies. In view of the findings of the present study, they could facilitate the transition from the utilization of concrete metaphors to the employment of unconventional metaphors as well as enabling the conceptualization of diverse present and future scenarios. This approach has the potential to promote enhanced emotional regulation, decision-making, self-cohesion. At the same time, this possible intervention strategy could enhance the ability to imagine and savor the benefits of a drug-free future (Mian and Earleywine, 2022), thereby reducing temporal discounting, one of the main determinants of addiction and relapse (Bickel et al., 2014). As Thurnherr (2021) argued, the power of metaphorical frameworks—such as conceptualizing the self as a *garden*—may promote client reflection and facilitate change, thereby underscoring the importance of perceiving metaphors not solely as a window into psychopathology but also as a catalyst for transformation.

## Conclusion

In conclusion, the present study underlines how people with addictive disorders may concretize their experience by using ontological and movement metaphors. The pervasive utilization of these metaphorical clusters seems to be a simple and basic way to enter into an intersubjective relationship with the other and share the complexity of one's experience. Through concrete metaphors, patients can narrate their internal conflicts and express their emotional ambivalence, offering both representational clarity and emotional containment. When understood not merely as linguistic embellishments but as cognitive and relational tools, metaphor enable patients to formulate their sense of self during moments of vulnerability. This perspective opens up important clinical implications: metaphor use can serve as both an assessment lens and an intervention strategy, offering clinicians a powerful means to access, explore, and transform patients' lived experience within the therapeutic process of change and recovery.

## Data Availability

The data from this study contain sensitive information. In accordance with the guidelines of the ethics committee that approved the research, they are therefore made available only upon reasonable request to the corresponding author.
